# Genomic Signatures of Environmental Adaptation in *Castanopsis hainanensis* (Fagaceae)

**DOI:** 10.3390/plants14071128

**Published:** 2025-04-05

**Authors:** Sha Li, Xing Chen, Yang Wu, Ye Sun

**Affiliations:** Guangdong Key Laboratory for Innovative Development and Utilization of Forest Plant Germplasm, College of Forestry and Landscape Architecture, South China Agricultural University, Guangzhou 510642, China; 20222159005@stu.scau.edu.cn (S.L.); chenxinglf@163.com (X.C.); yangwuhcy@163.com (Y.W.)

**Keywords:** adaptive variation, *Castanopsis hainanensis*, climate change, genome resequencing, risk of non-adaptedness

## Abstract

As an endemic *Castanopsis* species on Hainan Island, *Castanopsis hainanensis* Merr. is uniquely adapted to tropical climatic conditions and occupies a relatively narrow habitat range. Given its long generation times, limited dispersal capacity, and ecological and economic importance, understanding the genomic processes shaping this dominant tree species is critical for conservation. Its adaptation to specialized habitats and distinct geographical distribution provide valuable insights into biodiversity challenges in island ecosystems. This study employs genome-wide single-nucleotide polymorphism (SNP) markers to investigate genetic structure, population dynamics, and adaptive variation. Analyses revealed weak genetic divergence among populations, suggesting high gene flow. Demographic reconstruction indicated a historical population bottleneck, consistent with MaxEnt modeling projections of future range contraction under climate change. Selective sweep and genotype–environment association (GEA) analyses identified SNPs strongly correlated with environmental variables, particularly moisture and temperature. Using these SNPs, we quantified the risk of non-adaptedness (RONA) across climate scenarios, pinpointing regions at heightened vulnerability. Gene Ontology (GO) enrichment highlighted the key genes involved in plant growth and stress adaptation. By integrating genomic and environmental data, this study establishes a framework for deciphering adaptive mechanisms of *C. hainanensis* and offers actionable insights for informed conservation strategies to mitigate climate-driven biodiversity loss.

## 1. Introduction

The present-day distribution of plant species is heavily influenced by historical climate fluctuations and major geological events [1]. To reduce the risk of population collapse from environmental threats, plants have developed remarkable adaptive capacities to thrive in specific habitats, with positive selection playing a pivotal role in driving these adaptations [2,3,4]. Such adaptive processes leave detectable signatures, known as selective sweeps, in genomic regions near the selected genes. In specific environments, climatic factors act as selective pressures, shaping plant adaptation over time [5]. Tropical trees, however, pose unique challenges for unraveling these evolutionary mechanisms due to their long generation times and complex population structures [6]. Understanding how such species sustain adaptive potential is critical for predicting ecosystem resilience, as foundation trees disproportionately shape forest biodiversity and stability [7].

For species with limited genomic resources, pinpointing the molecular basis of environmental adaptation and its selective drivers remains challenging [8]. Nevertheless, advances in the genome-wide resequencing of non-model species now enable researchers to address this gap more effectively [9,10,11]. In forest trees, understanding patterns of adaptive genetic variation and their evolutionary history is essential for predicting responses to environmental changes [12]. Genotype–environment association (GEA) analyses offer a powerful tool to identify loci under environmental selection [9,13]. Unlike phenotype-dependent methods, GEA requires no prior phenotypic data, making it broadly applicable to non-model species [9]. To overcome inherent limitations, integrating complementary approaches—such as selective sweep detection, demographic modeling, and functional annotation—has become a widely adopted strategy [13,14,15,16]. Ultimately, deciphering the genetic mechanisms of environmental adaptation is crucial for forecasting species’ capacity to survive rapid climate change and for guiding targeted conservation efforts [17].

*Castanopsis hainanensis* Merr. is an endemic tree species restricted to Hainan Island, China [18]. It primarily inhabits mountainous regions, including Bawang, Diaoluo, Jianfeng, and Yingge, where it thrives in tropical forests below 400 m [19]. Despite challenging climatic conditions characterized by high temperatures and heavy rainfall, *C. hainanensis* exhibits strong growth performance and remarkable adaptability, making it an ideal model for studying adaptive evolution mechanisms. Investigating how environmental factors shape the adaptation of tree species like *C. hainanensis* could offer critical insights into forest ecosystem resilience and stability under climate change [20,21].

In this study, we leveraged whole-genome resequencing data to explore the genetic basis of environmental adaptation in *C. hainanensis*. Our objectives were threefold: (1) to characterize the population structure of *C. hainanensis*, (2) to reconstruct its evolutionary history, and (3) to identify genomic signatures of natural selection. The high-quality resequencing data and genome-wide SNPs generated here not only provide a foundational framework for elucidating adaptive genomic mechanisms, but also offer actionable insights to inform conservation strategies for this endemic species.

## 2. Results

### 2.1. Sequencing Quality

Whole-genome resequencing of 30 *C. hainanensis* individuals produced 310 Gb of raw sequencing data, with an average depth of 11.7145× per genome. Clean reads exhibited a 99.48% average alignment rate to the *Castanopsis tibetana* reference genome during quality-controlled mapping. After variant calling and stringent filtering steps, we obtained a total of 2,844,453 high-quality SNPs.

### 2.2. Population Structure 

**Figure 1:**
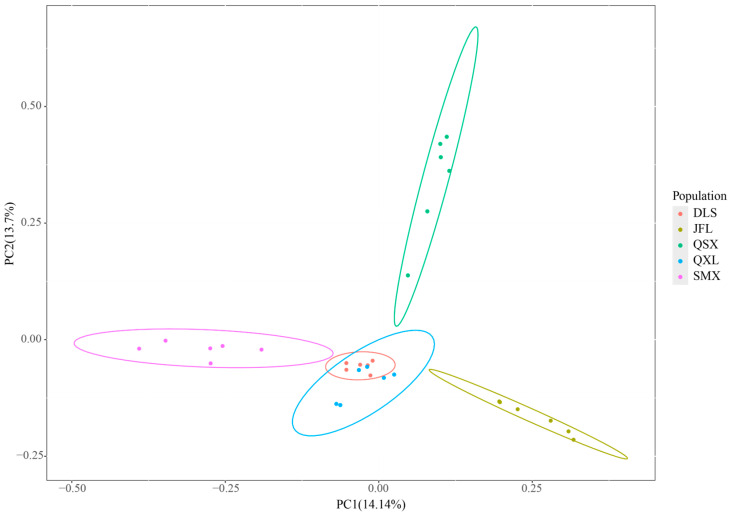
Principal component analysis (PCA) of all sampled *Castanopsis hainanensis* individuals. The first two principal components (PC1 and PC2) accounted for 14.14% and 13.7% of the total genetic variation, respectively.

**Figure 2:**
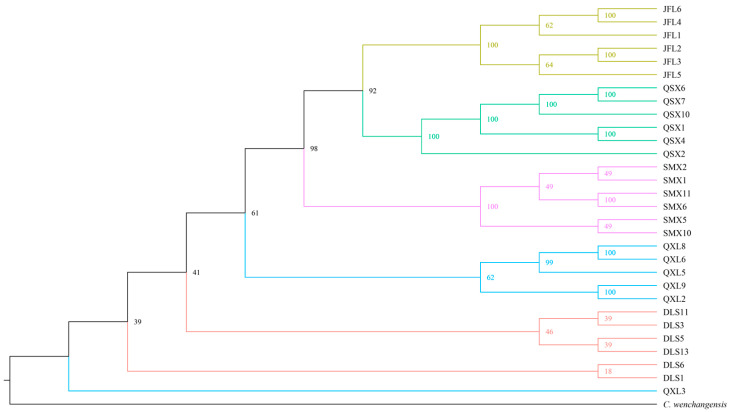
Maximum likelihood (ML) tree reconstructed from genome-wide SNPs of *Castanopsis hainanensis*.

**Figure 3:**
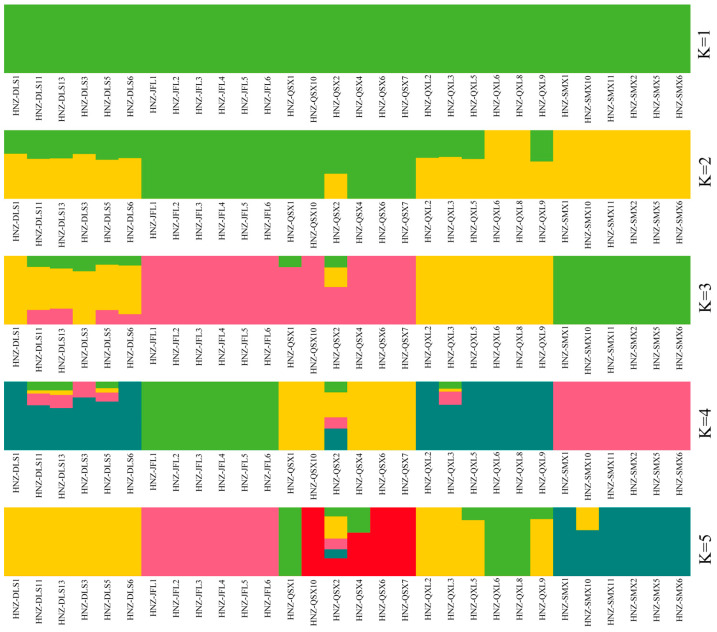
Genetic structure of *Castanopsis hainanensis* inferred using ADMIXTURE analysis. Individual ancestry proportions are shown for models assuming one to five ancestral clusters (K = 1–5).

**Figure A1:**
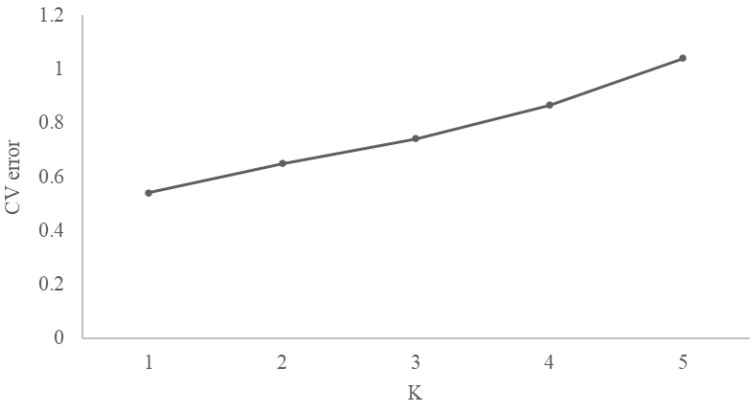
Cross-validation (CV) error curve for ADMIXTURE analysis of *Castanopsis hainanensis* as K values rise from 1 to 5.

Principal component analysis (PCA) revealed that individuals of *C. hainanensis* clustered approximately by geographical origin along the first two principal components, which collectively accounted for 14.14% and 13.7% of the genetic variation, respectively (Figure 1). The first principal component distinctly separated the JFL and SMX populations, while the second component differentiated QXS from others. However, the DLS and QXL populations showed overlapping clustering, indicating limited genetic differentiation. Consistent with PCA, the maximum likelihood (ML) tree (Figure 2) resolved five clades corresponding to geographical populations, though some DLS and QXL individuals exhibited ambiguous placement. Cross-validation errors in ADMIXTURE analysis increased incrementally as K values rose from 1 to 5 (Figure A1), suggesting minimum population genetic structure. Genetic structure analysis at K = 4 (Figure 3) further subdivided JFL, QSX, and SMX into distinct ancestral clusters, while DLS and QXL individuals remained admixed. Integrating the PCA, ML tree, and genetic structure results, we propose that a genetic model incorporating four ancestral components best explains the population architecture of *C. hainanensis*.

### 2.3. Demographic History

**Figure 4:**
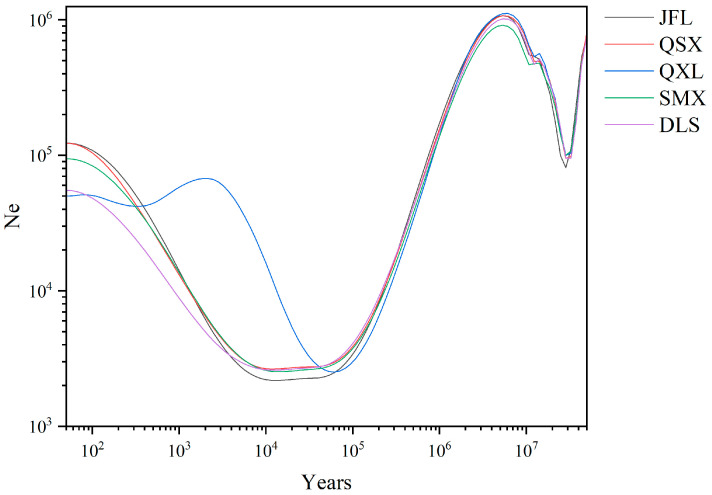
Temporal dynamics of effective population size (*Ne*) across five *Castanopsis hainanensis* populations.

The effective population size (*Ne*) trajectories of five *C. hainanensis* populations are illustrated in Figure 4. All populations displayed broadly concordant demographic patterns. A sharp decline in *Ne* occurred between approximately 40 and 20 million years ago (Mya), culminating in a population bottleneck. Following this, *Ne* gradually increased, peaking around 6 Mya. Subsequently, all populations underwent a rapid contraction before rebounding with a renewed expansion. Notably, the JFL and QSX populations experienced the mildest decline, whereas QXL suffered the most severe reduction. Diverging from the others, QXL began expanding around 0.05 Mya, reaching a modest peak before stabilizing. In contrast, the remaining populations resumed expansion later, at approximately 0.01 Mya.

### 2.4. Changes in Potential Habitats

**Figure A2:**
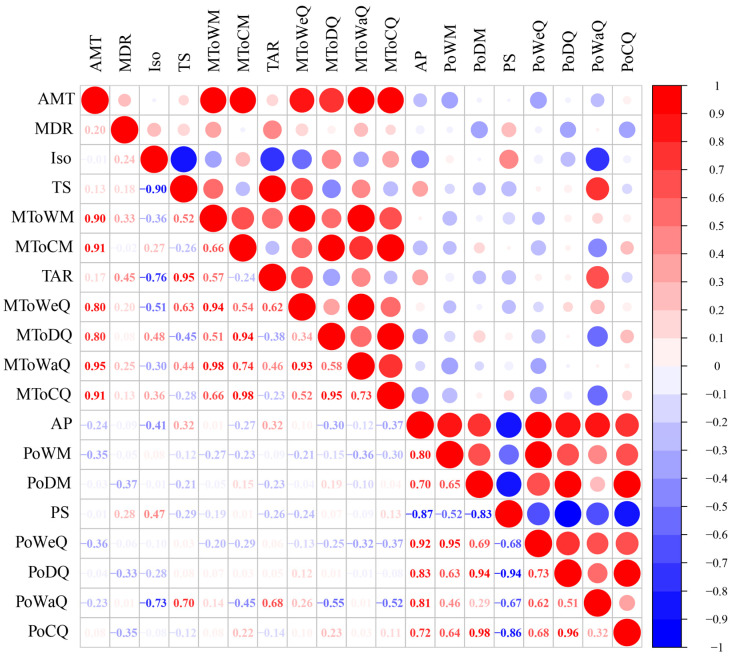
Pearson correlations among nineteen bioclimatic variables.

**Figure A3:**
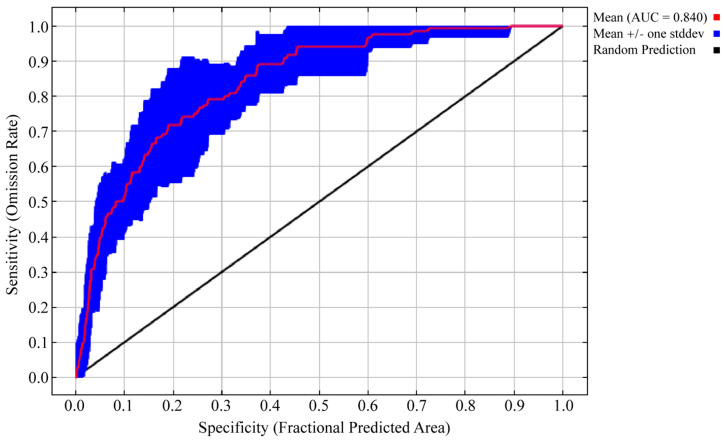
Area under the receiver operating characteristic (ROC) curve (AUC) of the MaxEnt model for *Castanopsis hainanensis*.

A jackknife test analysis, based on Pearson correlation coefficients between environmental variables (Figure A2), identified the following key predictors of *C. hainanensis* geographic distribution: BIO2 (Mean Diurnal Range), BIO3 (Isothermality), BIO6 (Minimum Temperature of Coldest Month), BIO8 (Mean Temperature of Wettest Quarter), BIO13 (Precipitation of Wettest Month), BIO15 (Precipitation Seasonality), and BIO18 (Precipitation of Warmest Quarter). The MaxEnt model for *C. hainanensis* achieved high performance, with an area under the receiver operating characteristic (ROC) curve (AUC) of 0.84 (Figure A3), demonstrating robust predictive accuracy.

**Figure 5:**
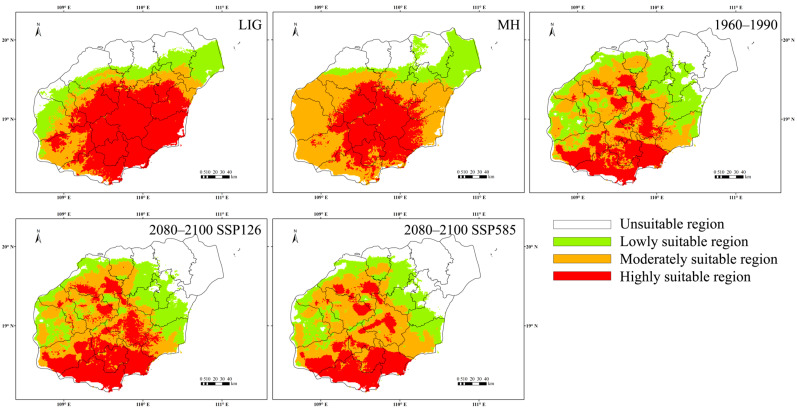
Projected shifts in suitable habitats for *Castanopsis hainanensis* across key climatic periods: LIG (Last Interglacial), MH (mid-Holocene), 1960–1990 (current baseline), and 2081–2100 (future long-term projections under SSP scenarios).

The potential habitat area of *C. hainanensis* expanded from the Last Interglacial (LIG) to the mid-Holocene (MH), but declined significantly from the MH to the present (1960–1990), with highly suitable area becoming increasingly fragmented. Currently, its potential habitat is concentrated primarily in the Bawang, Diaoluo, Jianfeng, and Yingge mountain regions, consistent with its known distribution. The total potential habitat spans approximately 2.35 × 10^4^ km^2^, including 6.42 × 10^3^ km^2^ of highly suitable and 9.56 × 10^3^ km^2^ of moderately suitable area. While the habitat of *C. hainanensis* has contracted overall according to model projections, the reduction in total area is not pronounced. Future scenarios predict habitat loss of approximately 1% by 2080 to 2100 under the low-emission scenario (SSP126). In contrast, under the highest-emission scenario (SSP585), habitat reduction could reach up to 5% (Figure 5 and Table A1).

### 2.5. Selective Sweeps

**Figure 6:**
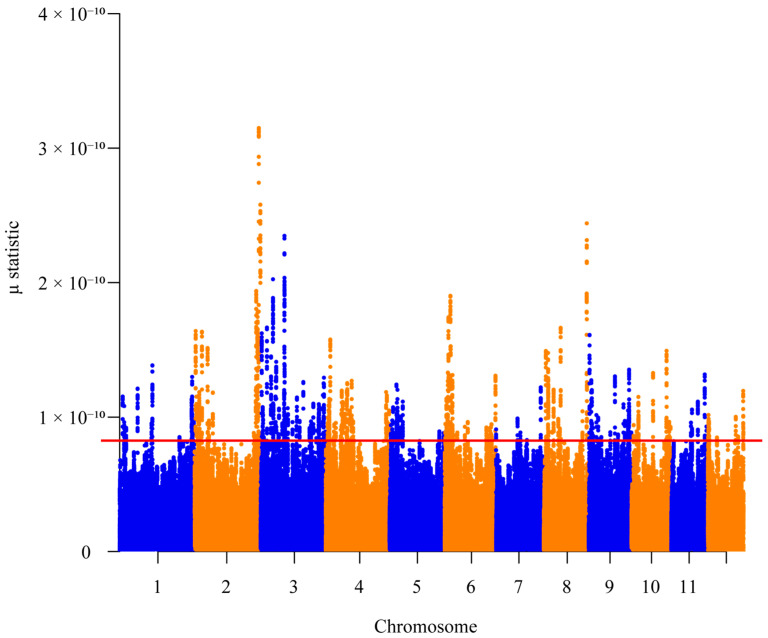
Manhattan plot illustrating genome-wide selective sweeps in *Castanopsis hainanensis*. The μ statistic is plotted on the y-axis against genomic positions across chromosomes on the x-axis. Each solid circle represents an individual SNP, with elevated μ values highlighting regions under strong selection. A red horizontal line denotes the significance threshold (top 0.05% of μ values).

**Figure 7:**
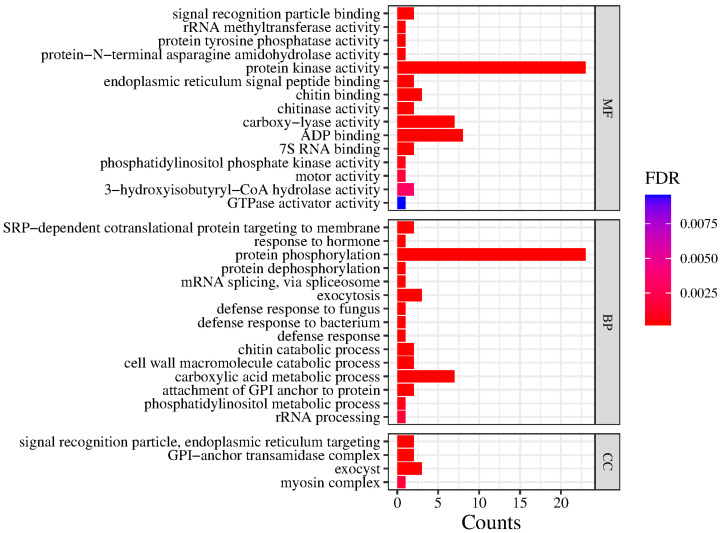
Functional enrichment of genes within selective sweep regions in *Castanopsis hainanensis*. Bar heights indicate the number of enriched genes assigned to Gene Ontology (GO) categories: biological process (BP), cellular component (CC), and molecular function (MF).

A total of 5934 SNPs were detected within selective sweep regions (Figure 6). The Gene Ontology (GO) enrichment analysis revealed 34 significantly enriched terms, with 15, 4, and 15 terms assigned to the biological process (BP), cellular component (CC), and molecular function (MF) categories, respectively (Figure 7). Key enriched biological processes included protein phosphorylation, the carboxylic acid metabolic process, exocytosis, GPI-anchor attachment, cell wall macromolecule catabolism, SRP-dependent cotranslational protein targeting, and chitin catabolism. Enriched cellular components comprised the exocyst, the GPI-anchor transamidase complex, signal recognition particles, endoplasmic reticulum targeting components, and the myosin complex. Molecular functions were predominantly linked to protein kinase activity, ADP binding, carboxy-lyase activity, chitin binding, endoplasmic reticulum signal peptide binding, 7S RNA binding, signal recognition particle binding, chitinase activity, and 3-hydroxyisobutyryl-CoA hydrolase activity. BLAST v1.4.0 analysis against the NCBI database identified candidate adaptive genes in *C. hainanensis* associated with abiotic stress response (e.g., *FERONIA*, *RPP13-like*, *WIN1*, and *RML1A-like*), growth and development regulation (e.g., *TMK4*, *FERONIA*, and *RML1A-like*), and disease resistance (e.g., *At3g47570*, *EIX2*, *STH-21*, *PR-4*, *WIN1*, *EXO70B1*, *RGA3*, *At4g27220*, *RPP13-like*, and *At1g58400*).

### 2.6. Genetic–Environment Association Analysis

**Figure 8:**
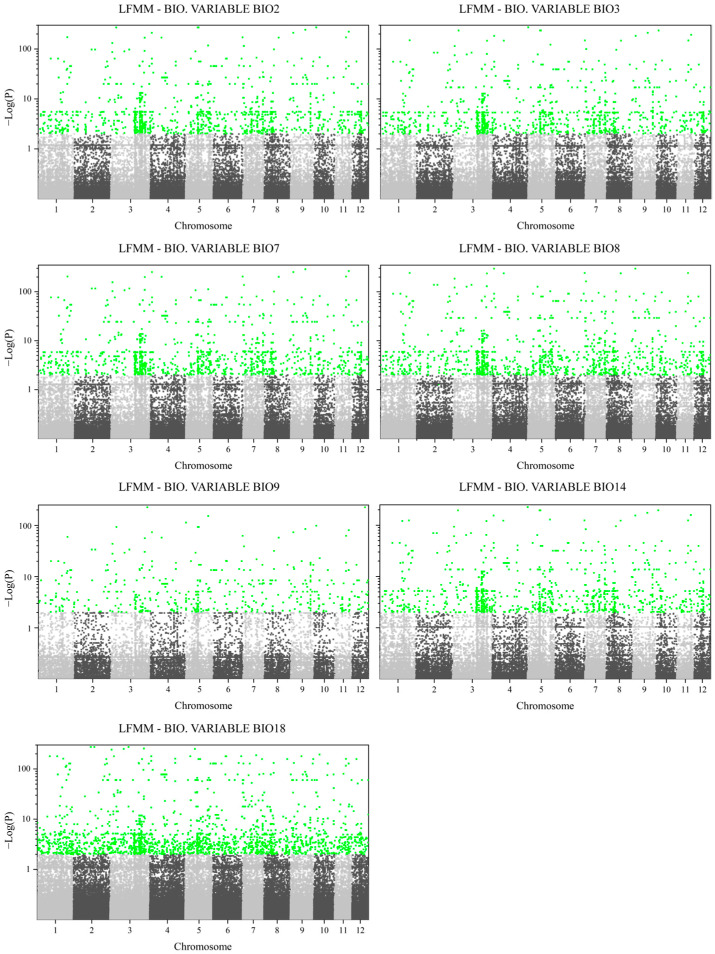
Manhattan plot generated by the Latent Factor Mixed Model (LFMM) analysis, depicting genome-wide associations between SNPs and environmental variables. Green points represent SNPs with statistically significant correlations (False Discovery Rate (FDR) < 0.01).

**Figure A4:**
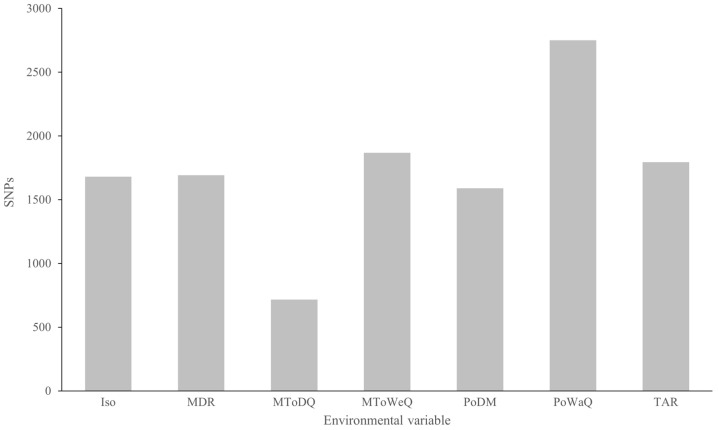
Distribution of 3263 SNP–environment associations (False Discovery Rate (FDR) < 0.01) identified via Latent Factor Mixed Model (LFMM) across environmental variables.

**Figure A5:**
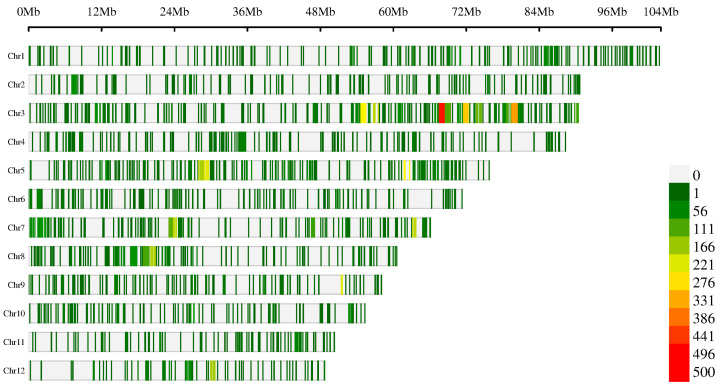
Distribution of candidate adaptive SNPs across 12 chromosomes of *Castanopsis hainanensis* (1Mb window analysis).

A total of 3263 SNPs demonstrated significant associations with one or more environmental variables in the Latent Factor Mixed Model (LFMM model; K = 4 latent factors) (Figure 8). Among the tested climatic factors, Precipitation of Warmest Quarter (BIO18) emerged as the most influential, correlating with 2750 SNPs. This was followed by Mean Temperature of Wettest Quarter (BIO8; 1868 SNPs), Temperature Annual Range (BIO7; 1794 SNPs), Mean Diurnal Range (BIO2; 1693 SNPs), and Isothermality (BIO3; 1680 SNPs), with variable-specific SNP counts detailed in Figure A4. The genomic distribution analysis (1 Mb window size) revealed an uneven clustering of candidate adaptive SNPs across chromosomes (Figure A5). Chromosome 3 harbored the highest number of loci (694 SNPs), while chromosome 12 contained the fewest (132 SNPs). A Pearson’s correlation analysis confirmed no significant relationship between chromosome size and SNP abundance (*p* > 0.05). Notably, two pronounced SNP hotspots were identified in the latter half of chromosome 3, suggesting localized regions of adaptive genetic variation.

**Figure 9:**
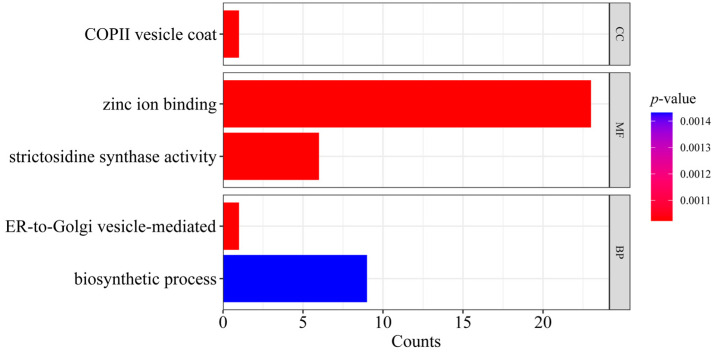
Gene Ontology (GO) enrichment analysis of *Castanopsis hainanensis* genes harboring putative adaptive SNPs. Bar heights indicate the number of enriched genes (counts) across three functional categories: biological process (BP), cellular component (CC), and molecular function (MF).

We identified candidate genes harboring at least one putative adaptive SNP. The GO enrichment analysis (False Discovery Rate (FDR) < 0.01) revealed significant enrichment in five GO terms across three categories (Figure 9). In biological processes, candidate genes were associated with biosynthetic processes and ER-to-Golgi vesicle-mediated transport, which may facilitate the synthesis and intracellular transport of critical biomolecules, reflecting their critical role in cellular homeostasis and environment adaptability. For cellular components, the enriched terms of COPII vesicle coat suggests its importance in maintaining precise intracellular transport mechanisms in *C. hainanensis*. Within molecular functions, zinc ion binding and strictosidine synthase activity were predominant, implicating these functions in potential regulatory networks and genetic information processing, which may underpin adaptation to complex physiological conditions. A BLAST comparison with the Arabidopsis protein database identified seven *C. hainanensis* genes potentially linked to environmental adaptation on Hainan Island. These include stress resistance-related genes (e.g., *MMS21*, *PCRK1*, *SEC23*, and *SYNTHASE-LIKE 6*) and growth and development regulators (e.g., *H3 lysine-9 specific SUVH5*, and *SYNTHASE-LIKE 5*). These loci may contribute to resilience against the unique tropical monsoon climate and diverse biotic pressures on Hainan Island.

### 2.7. Risk of Non-Adaptedness (RONA)

**Figure 10:**
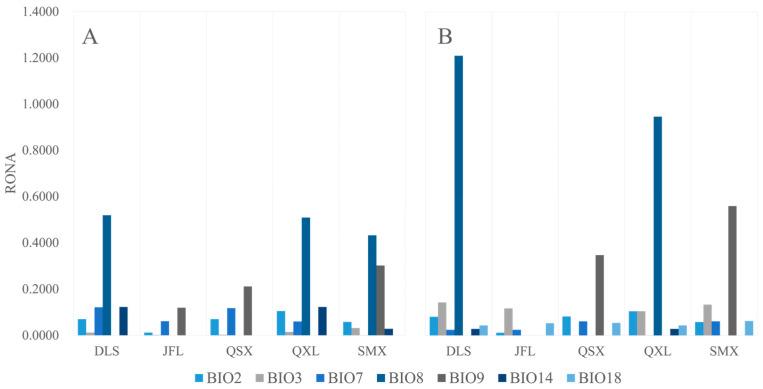
Risk of non-adaptedness (RONA) estimates for seven bioclimatic variables across *Castanopsis hainanensis* under different climate scenarios (2081–2100): (**A**) SSP126 (low emissions) and (**B**) SSP585 (high emissions). Bioclimatic variables (BIOs) are defined as follows: BIO2, Mean Diurnal Range; BIO3, Isothermality; BIO7, Temperature Annual Range; BIO8, Mean Temperature of Wettest Quarter; BIO9, Mean Temperature of Driest Quarter; BIO14, Precipitation of Driest Month; BIO18, Precipitation of Warmest Quarter.

By leveraging established genotype–environment relationships and climate-associated SNPs, we calculated the RONA for seven environmental variables under the SSP126 and SSP585 future climate scenarios (2080–2100), designated as the optimal and worst-case projections, respectively, for *C. hainanensis* (Table A2 and Table A3). Among the variables analyzed, Precipitation of Warmest Quarter exhibited the strongest genomic signature, with 1936 associated SNPs (average r^2^ = 0.2565). This was followed by Mean Temperature of Wettest Quarter (1418 SNPs, average r^2^ = 0.2617), Temperature Annual Range (1393 SNPs, average r^2^ = 0. 2359), Mean Diurnal Range (1342 SNPs, average r^2^ = 0. 2856), and Isothermality (1326 SNPs, average r^2^ = 0. 2379). These results align closely with the genotype–environment association analyses, reinforcing the robustness of the findings. Notably, the DLS population demonstrated higher RONA values (Figure 10 and Table A2 and Table A3), suggesting greater vulnerability under the projected climate conditions.

## 3. Discussion

PCA analysis supports the geographical division of *C. hainanensis* into four distinct genetic groups. Clear differentiation is observed among populations from JFL, QSX, and SMX, while populations from DLS and QXL exhibit minimal divergence. These findings align with the results from the ML tree and ADMIXTURE analyses, highlighting both population differentiation and genetic admixture in *C. hainanensis*. Further analysis integrating geographical data suggests that the complex mountainous and riverine terrain within the Hainan Tropical Rainforest significantly shapes the species’ genetic structure [22]. Such barriers likely restrict migration and gene flow, fostering unique genetic characteristics over prolonged evolutionary periods [23,24]. Populations from JFL, QSX, and SMX, separated by considerable geographic distances, have developed distinct genetic lineages. In contrast, SMX, DLS, and QXL—clustered in closer proximity—show facilitated gene flow. Notably, SMX lies within the Wuzhi Mountains, a natural barrier that may limit gene exchange with other populations, maintaining its genetic independence [25,26]. The minimal differentiation between DLS and QXL may stem from their shared low-altitude hilly terrain or interspecific gene flow in overlapping zones [27]. Habitat fragmentation within the Hainan tropical rainforest exacerbates constraints on gene flow. For effective conservation, management strategies should prioritize genetic connectivity over strict geographical boundaries [28]. This study underscores that geographical isolation does not invariably drive genetic differentiation. Conservation planning must therefore integrate interactions between habitat geography and species-specific gene flow dynamics informed by genetic structure analyses to guide scientifically robust ecological management.

Historical climate shifts and vicariance events profoundly shaped the distribution and evolutionary trajectory of *C. hainanensis* [1,29,30,31]. The initial decline at approximately 40 Mya in the effective population size (*Ne*) of *C. hainanensis* aligns with the Eocene–Oligocene transition, a period of dramatic geological changes on Hainan Island [32,33,34]. Intensified East Asian monsoons, driven by Indian Plate subduction, amplified seasonal aridity and precipitation variability [35,36,37], while paleo topographic uplift fragmented habitat, isolating populations into high-elevation refugia [38]. Comparable demographic contractions during this period have been documented in other *Castanopsis* species [39]. By the mid-Miocene Climatic Optimum, global temperatures rose 3–4 °C above modern levels [40], coinciding with stabilized topography on Hainan Island. This enabled renewed gene flow among *C. hainanensis* populations, culminating in a historical peak in effective population size of ~6 Mya. During the Last Glacial Maximum period (LGM), cooling temperatures triggered widespread habitat loss [29,30,31], driving another population bottleneck. Post-LGM recovery of the effective population size was likely enabled by refugia in the central highlands of Hainan Island [29]. Despite these fluctuations, contemporary populations remain demographically stable. Jackknife tests identified Isothermality (BIO3) and Precipitation Seasonality (BIO15) as key climatic drivers, underscoring that *C. hainanensis* is sensitive to seasonal temperature and rainfall shifts. Distribution modeling reveals that high-suitability habitats since the Holocene have clustered in southern mountainous regions (e.g., the Bawang, Diaoluo, and Jianfeng Mountains), aligning with the species’ current range and highlighting the critical role of topographic heterogeneity in survival. However, these habitats have contracted markedly, with remaining high-suitability zones fragmented by the steep elevational gradient in Hainan’s central highlands. This geological configuration promotes vertical vegetation stratification adapted to elevation-driven microclimates, while restricting dispersal between altitudinal zones. Future projections predict further range contraction, particularly under high-emission scenarios (SSP585). While overall warming and humidification are anticipated [41], southern low-latitude regions of Hainan Island may retain stable dry–wet seasonality, preserving localized pockets of suitability. Nevertheless, habitat fragmentation and reduced connectivity—exacerbated by natural topographic barriers—pose significant threats to long-term population resilience.

Trees inhabit diverse environments, showcasing remarkable ecological adaptability shaped by local conditions [4,9]. For *C. hainanensis* on Hainan Island, temperature and precipitation are critical drivers of distribution and growth [42], defining the region’s tropical climate. These factors also underpin the species’ adaptive evolution, as plants frequently respond to environmental shifts through positive selection—a process that rapidly fixes advantageous alleles, leaving detectable genetic signatures (selective sweeps) in regions linked to adaptive traits [43,44,45]. Identifying these genomic regions and their functional roles is key to unraveling how *C. hainanensis* adapts to the island’s unique conditions. Selective sweep detection and genotype–environment association (GEA) analyses have pinpointed genes critical to environmental adaptation. These genes primarily govern stress responses and development regulation. For instance, the genes *FERONIA*, *RPP13-like*, and *RML1A-like* enhance heat tolerance [46,47,48], while *FERONIA*, *WIN1*, and *MMS21* bolster drought resistance [49,50,51]. The genes *FERONIA*, *PR-4*, and *SYNTHASE-LIKE 6* mitigate abiotic stresses such as heavy metals, salinity, and oxidative damage [52,53,54]. The genes *At3g47570*, *EXO70B1*, *RGA3*, *RPP13-like*, and *PCRK1* are integral components of plant immune defense [55,56,57,58,59], and likely aid survival in Hainan’s hot and humid climate, where pathogen risks are elevated. The genes *SEC23*, *H3 lysine*—*9 specific SUVH5*, and *SYNTHASE*—*LIKE 5* regulate leaf development and flowering in plants [60,61,62], and could ensure developmental stability in *C. hainanensis* under fluctuating humidity and temperature. Historically, climate and geological shifts have reshaped *Castanopsis* habitats, driving novel selective pressures [39,63]. Understanding the genetic adaptation of *C. hainanensis* to its tropical monsoon environment—marked by high heat and humidity—can inform targeted conservation strategies. Protecting habitats, monitoring genetic diversity, and enabling assisted migration are crucial for safeguarding the species. Moreover, identifying adaptive genes will help predict resilience to future climate challenges, ensuring the long-term survival of *C. hainanensis* amid rapid environmental change.

RONA quantifies the theoretical allele frequency shifts needed at climate-linked loci for populations to track projected environmental changes [13]. For *C. hainanensis*, RONA was modeled under two shared socioeconomic pathways (SSP126: low emissions; SSP585: high emissions), using seven key climate variables. Precipitation of Warmest Quarter emerged as the strongest SNP-associated driver of adaptation, followed by Mean Temperature of Wettest Quarter, Temperature Annual Range, Mean Diurnal Range, and Isothermality—factors representing the foremost selective pressures under climate change. Elevated RONA values under SSP585 suggested increased extinction risks for *C. hainanensis*, since its slow generational turnover may lag behind the pace of allele frequency shifts required to cope with accelerating warming [64]. Populations with high RONA values (e.g., DLS and QSX) face amplified vulnerability due to limited dispersal and fragmented habitats. Although gene flow from fitter populations could theoretically alleviate selective pressure by introducing adaptive alleles [65], the species’ restricted endemic range and habitat fragmentation may severely constrain genetic connectivity, leading to a conflict between migration dynamics and selective pressures that exacerbates maladaptation [66]. Future studies should integrate landscape genetics approaches to assess how topography modulates gene flow and its implications for RONA prediction. These efforts can be combined with real-time monitoring networks to track allele frequency dynamics, alongside microhabitat changes, enabling adaptive management through ecological niche forecasting.

## 4. Materials and Methods

### 4.1. Sampling, Library Preparation, and Sequencing

**Figure 11:**
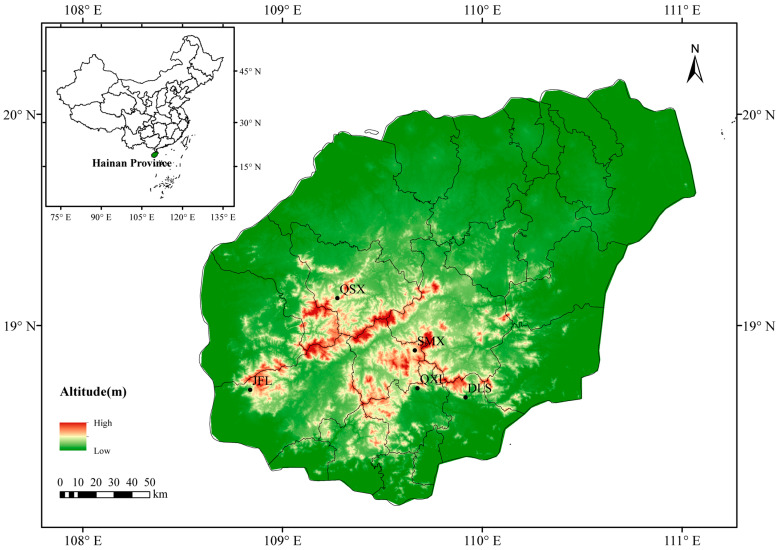
Sampling locations of *Castanopsis hainanensis*. Population codes are the same as in Table 1.

We sampled 30 *C. hainanensis* individuals from 5 populations (Table 1; Figure 11), representing the current natural range of this species [67,68]. Fresh leaves were collected from mature trees spaced at least 20 m apart to minimize genetic relatedness and were immediately field-dried using silica gel to prevent tissue degradation.

DNA extraction and library preparation were performed by BGI Genomics (China). Genomic DNA was fragmented via sonication to 350 base pairs (bp), followed by end repair, A-tailing, and the ligation of Illumina-compatible adapters. The libraries were then amplified by PCR, purified, and assessed for size distribution using an Agilent2100 Bioanalyzer. Quantification was performed via real-time PCR. Following quality control (QC), qualified DNA libraries were mechanically sheared using a Covaris ultrasonicator to ensure uniform fragment size (~350 bp). Fragments were size-selected using magnetic beads, end-repaired, and adenylated at 3′ ends to enable adapter ligation. Ligated products were cyclized and amplified via linear isothermal Rolling-Circle Replication, followed by DNA NanoBall (DNB) synthesis. Paired-end sequencing (150 bp reads) was performed on the DNBSEQ-T7 sequencing platform (MGI, China). Raw reads were processed using SOAPnuke (developed by BGI) to remove low-quality reads and adapter sequences [69].

### 4.2. SNP Calling

The resultant high-quality reads were aligned against the *Castanopsis tibetana* genome [70] using the BWA-MEM2 GUI Wrapper in TBtools v1.120 [71]. The aligned reads were subsequently sorted, and duplicate reads were marked using the SAMtools GUI Wrapper (also within TBtools v1.120). Alignment quality was evaluated using Qualimap v2 software [72], followed by SNP calling via the BCFtools GUI Wrapper in TBtools v1.120. Variants were filtered using stringent parameters: –g3 –G10 –e INFO/DP < 8 || INFO/DP>250 || %QUAL < 10 || (RPB < 0.1 && %QUAL < 15) || (AC < 2 && %QUAL < 15) || INFO/MQ < 30 || MQSB <= 0.1. This step yielded 2,207,442,773 SNPs. Further refinement was performed using PLINK v1.9 [73] to exclude SNPs with a genotype missing rate > 0.1 and a minor allele frequency (MAF) < 0.05.

### 4.3. Population Structure Analyses

The SNP data were filtered and pruned for linkage disequilibrium using PLINK v1.9 with the following command: indep-pairwise 50 10 0.1. A total of 2,844,453 high-quality SNPs were selected for subsequent analysis. Principal component analysis (PCA) was conducted using PLINK v1.9 [73], and the results were visualized using the R package “ggplot2” [74]. The hierarchical population structure was estimated using ADMIXTURE v1.3.0 [75], with the number of clusters (K) ranging from 1 to 5. The cross-validation (CV) errors were calculated for each K value. The population genetic structure matrix was constructed based on the individual genetic composition coefficient (Q) and visualized using Admixture Q Matrix Viz in TBtools v1.120 [71]. A maximum likelihood (ML) tree was constructed using RAxML v8 [76] with the GTRGAMMA model and 1000 bootstrap replicates to explore the genetic relationships among individuals. *Castanopsis wenchangensis* G. A. Fu et C. C. Huang was used as the outgroup. The resulting tree was finally visualized using Figtree software v1.4.4 (https://tree.bio.ed.ac.uk/software/figtree/ accessed on 15 February 2025).

### 4.4. Demographic History Analyses

We used the sequential Monte Carlo approach implemented in SMC++ [77] to evaluate the effective population size (*Ne*) over historical time. SMC++ can jointly infer population size histories and divergence times. It incorporates a novel spline regularization scheme that greatly reduces estimation error [77]. A VCF (Variant Call Format) file was converted into a specific input format recognizable by SMC++ when using the vcf2smc script (https://github.com/popgenmethods/smcpp?tab=readme-ov-file#vcf2smc accessed on 12 November 2024). The simulation was performed with a mutation rate of 8.21 × 10^−10^ [39]. The dynamic changes in the effective population size were visualized using the plot function in SMC++.

### 4.5. Species Distribution Model

The occurrence data of *C. hainanensis* were sourced from field investigations, herbarium records from the Global Biodiversity Information Facility (http://www.gbif.org), and the Chinese Virtual Herbarium (http://www.cvh.ac.cn/). For each location, nineteen bioclimatic variables were retrieved from the WorldClim v2.1 database (https://worldclim.org) at a resolution of 30 arc-seconds. To avoid overfitting the species distribution model (SDM) due to multicollinearity, Pearson correlation coefficients between the environmental variables were calculated using ENMtools v2.0 [78]. Variables with an absolute correlation coefficient greater than 0.8 were removed, prioritizing retention based on their percent contribution to a jackknife test [79,80]. The MaxEnt v3.4.4 [79] model was utilized to run SDMs for five periods: the Last Interglacial (LIG), mid-Holocene (MH), present (1960–1990), and future long-term (2081–2100). In the MaxEnt modelling, 25% of the distribution data were designated as the test dataset, while the remaining data served as the training dataset. The regularization multiplier was set to 2 to prevent over-complexity or overfitting [81], and the maximum number of iterations was set to 5000 to ensure model converge. Model performance was evaluated using the area under the receiver operating characteristic (ROC) curve (AUC). Finally, the SDM results were visualized, and suitable habitat areas were inferred using ArcGIS v10.4 (https://www.esri.com/en-us/arcgis/products/index accessed on 27 December 2024).

### 4.6. Detection of Selective Sweeps

Genomic regions under recent and strong adaptation in *C. hainanensis* were identified using RAiSD [82], a tool that detects selective sweeps by analyzing three distinct signatures: localized reduction in polymorphisms; shifted site frequency spectrum (SFS) toward low- and high-frequency-derived variants; and a specific linkage disequilibrium (LD) pattern characterized by elevated LD on the same side of a beneficial mutation and reduced LD between the loci flanking the mutation. We calculated the μ statistic using the default settings and defined genomic windows with the top 0.05% μ values as the putative selective sweep regions. We conducted Gene Ontology (GO) enrichment on candidate regions using GOWINDA [83]. The *p*-values were corrected for multiple testing using the Benjamini–Hochberg FDR method [84], and GO terms with FDR < 0.01 were considered as significantly enriched.

### 4.7. Genetic–Environment Association (GEA) Analysis

To identify SNPs significantly associated with specific environmental variables, we conducted a genome-wide association analysis [13]. The Latent Factor Mixed Model (LFMM) approach, implemented in the R package “LEA” [85], was used to evaluate these associations while accounting for population structure. Multiple testing was addressed by converting the *p*-values obtained from LFMM into q-values using the R package “qvalue”. SNPs with an FDR of less than 1% were considered as candidate loci. These candidate loci were then subjected to genomic annotation. To elucidate protein function information, the BLAST v1.4.0 (https://blast.ncbi.nlm.nih.gov/Blast.cgi accessed on 14 February 2025) was used to compare the genes associated with these SNPs with the Arabidopsis thaliana proteome.

### 4.8. Risk of Non-Adaptedness (RONA) Under Future Climatic Scenarios

We evaluated the RONA for *C. hainanensis* under future climate scenarios using a python implementation of "Risk of non Adaptedness" method [13]. RONA quantifies the average discrepancy between current and projected future allele frequencies, serving as a proxy for the magnitude of genetic change required for the species to adapt to future climatic conditions [13]. A linear relationship between the allele frequencies of environmentally associated loci and present-day climate conditions was established. Seven uncorrelated environmental variables, expected to shift under future climate scenarios, were selected to minimize redundancy and model complexity. Using the derived linear model, we predicted allele frequencies in 2090 across environmental gradients under two Shared Socioeconomic Pathways (SSPs): SSP126 (low emission) and SSP585 (high emission). RONA was computed per individual using candidate adaptive SNPs identified via LFMM analysis.

## 5. Conclusions

In this study, the integrative analysis of genetic structure revealed moderate population subdivision within *C. hainanensis*, though genetic admixture was still observed among geographically adjacent populations. Temporal changes in the effective population size of *C. hainanensis* aligned with historical climatic fluctuations and vicariance events, while species distribution modeling projected a contraction of suitable habitat under future climate change scenarios, highlighting the urgency of implementing in situ conservation measures. Through genome-wide scans for positive selection signals, we identified SNPs exhibiting significant associations with environmental variables and characterized candidate adaptive genes primarily related to growth regulation, developmental processes, and stress resistance mechanisms. These findings advance our understanding of how *C. hainanensis* has evolutionarily adapted to tropical environments characterized by high-humidity and high-temperature conditions. Future studies could enhance vulnerability assessments by integrating adaptive genetic variation with ecological niche modeling, thereby enabling more precise predictions of species resilience under shifting climatic regimes. Such integrative approaches will prove critical for designing targeted conservation strategies that address both the genomic and ecological dimensions of species persistence.

## Figures and Tables

**Table 1:** Sampling location information for *Castanopsis hainanensis*.

Population	Sampling Location	Elevation (m)	Longitude (°)	Latitude (°)
DLS	Diaoluoshan Mountain, Lingshui County	377	109.9160	18.6598
JFL	Jianfengling Mountain, Ledong County	283	108.8379	18.6957
QSX	Qingsong Village, Baisha County	396	109.2745	19.1304
QXL	Qixianling Mountain, Baoting County	264	109.6759	18.7034
SMX	Shuiman Village, Wuzhishan City	658	109.6629	18.8830

## Data Availability

The data that support the findings of this study have been deposited into CNGB Sequence Archive (CNSA) of China National GeneBank DataBase (CNGBdb) with accession number CNP0006931.

## Abbreviations

The following abbreviations are used in this manuscript:

AUC	Area under the receiver operating characteristic (ROC) curve
BIO	Bioclimatic variables
CV	Cross-validation
CVH	Chinese Virtual Herbarium
FDR	False Discovery Rate
GEA	Genotype–environment association
GO	Gene Ontology
LD	Linkage disequilibrium
LFMM	Latent Factor Mixed Model
LIG	Last interglacial
MAF	Minor allele frequency
MH	Mid-Holocene
ML	Maximum likelihood
Mya	Million years ago
NCBI	National Center for Biotechnology Information
Ne	Effective population size
PCA	Principal component analysis
RONA	Risk of non-adaptedness
SDM	Species distribution model
SNP	Single-nucleotide polymorphism
SSP	Shared Socioeconomic Pathway
VCF	Variant Call Format

## Appendix A

**Table A1:** Potential habitat area of *Castanopsis hainanensis* across key climatic periods: LIG (Last Interglacial), MH (mid-Holocene), present (1960–1990), and 2081–2100 (future long-term projections under SSP scenarios).

Period	Unsuitable Region (km^2^)	Low-Suitability Region (km^2^)	Moderately Suitable Region (km^2^)	Highly Suitable Region (km^2^)
LGM	3845.14	4411.81	11,892.36	8888.89
MH	4929.17	5463.89	5375.7	13,269.44
Present	5746.53	7545.83	9564.58	6420.14
2080–2100 (SSP126)	5974.3	6909.03	8843.05	7550.69
2080–2100 (SSP585)	7030.56	6754.86	9775.69	5715.97

**Table A2:** RONA results for *Castanopsis hainanensis* populations under SSP126 (2080–2100) based on seven environmental factors.

Variable	bio2	bio3	bio7	bio8	bio9	bio14	bio18
SNPs	1342	1326	1393	1418	630	1186	1936
DLS	0.0699	0.0120	0.1217	0.5188	0.0000	0.1223	0.0578
JFL	0.0117	0.0018	0.0614	0.0000	0.1192	0.0000	0.0530
QSX	0.0699	0.0024	0.1177	0.0000	0.2115	0.0000	0.0000
QXL	0.1049	0.0144	0.0602	0.5084	0.0000	0.1223	0.0000
SMX	0.0583	0.0317	0.0000	0.4321	0.3016	0.0286	0.0616
Mean	0.0629	0.0125	0.0722	0.2918	0.1265	0.0547	0.0345
Min R^2^	0.0004	0.0007	0.0108	0.0020	0.0002	0.0230	0.0025
Max R^2^	0.9212	0.8253	0.8728	0.7412	0.7146	0.9713	0.7474
Average R^2^	0.2856	0.2379	0.2359	0.2617	0.2707	0.2139	0.2565

**Table A3:** RONA results for *Castanopsis hainanensis* populations under SSP585 (2080–2100) based on seven environmental factors.

Variable	bio2	bio3	bio7	bio8	bio9	bio14	bio18
SNPs	1342	1326	1393	1418	630	1186	1936
DLS	0.0807	0.1428	0.0242	1.2102	0.0000	0.0286	0.0434
JFL	0.0117	0.1167	0.0242	0.0000	0.0000	0.0000	0.0530
QSX	0.0819	0.0000	0.0614	0.0000	0.3469	0.0000	0.0543
QXL	0.1049	0.1048	0.0000	0.9461	0.0000	0.0285	0.0434
SMX	0.0583	0.1333	0.0614	0.0000	0.5592	0.0000	0.0625
Mean	0.0675	0.0995	0.0343	0.4313	0.1812	0.0114	0.0513
Min R^2^	0.0004	0.0007	0.0108	0.0020	0.0002	0.0230	0.0025
Max R^2^	0.9212	0.8253	0.8728	0.7412	0.7146	0.9713	0.7474
Average R^2^	0.2856	0.2379	0.2359	0.2617	0.2707	0.2139	0.2565
